# Management of complex patients in family medicine

**DOI:** 10.3325/cmj.2025.66.77

**Published:** 2025-02

**Authors:** Aleksandar Džakula, Dorja Vočanec

**Affiliations:** Center for Health Systems, Policies, and Diplomacy, Andrija Štampar School of Public Health, University of Zagreb School of Medicine, Zagreb, Croatia

## The increasing complexity in family medicine

Working in family medicine has never been easy or simple. However, with the growing scope of work, new diagnostic and therapeutic methods, and administrative procedures, the demands are becoming increasingly challenging. In addition to these pressures, there is a rising number of patients with complex and multidimensional needs – complex patients.

Complex patients present a significant challenge for primary health care physicians, especially for family medicine physicians. The phenomenon of complex patients is not new; it has always been a part of the reality of family medicine. Family medicine has long been a pioneer in providing comprehensive care to patients and their families, addressing all their needs across various dimensions. The challenges in this field arise from different aspects of care, including medical, social, organizational, and emotional factors (1). Successfully managing such patients requires an integrated approach and specific competencies.

A holistic approach in family medicine enables the recognition and management of not only medical but also psychosocial and social aspects of patients’ health. Furthermore, care for complex patients involves the coordination between different levels of health care (primary, secondary, and tertiary) and collaboration with other stakeholders, such as social workers, psychologists, and non-governmental organizations. In working with patients with complex needs, the ability to early recognize and manage both health and social risks is particularly important, as this can reduce the frequency of acute hospitalizations and improve long-term outcomes (2). However, caring for complex patients, especially the increasing number of them in family medicine practices, can also pose significant risks. A high patient load and administrative tasks often leave little room for a thorough assessment and care planning for complex patients.

Family medicine physicians frequently face limitations in accessing diagnostic tools, specialist consultations, or support from other professionals, which forces them to make decisions while shouldering great responsibility. Nevertheless, the key question remains: in what direction can family medicine evolve to meet the growing demands arising from the changing, increasing, and increasingly complex needs of patients (3)?

## The new yet familiar role of family medicine physicians in caring for complex patients

The role of family medicine physicians is to recognize the needs of patients and their families within the specific dynamics of the primary care community, as well as to provide guidance and support within that community. However, modern patient care needs and capabilities extend far beyond care limited to family and community settings.

Today, the family medicine physician is not only responsible for the patients and their families but also needs to coordinate various public services on which their patients depend. Whether it be issues related to employment rights and education or various certificates and documents required for transportation, property management, travel, or other legal matters – family physicians today take on an increasingly comprehensive role.

In this context, managing complex clinical and social needs may require specialized skills that are not always sufficiently covered in standard medical education. This gap is particularly evident among younger physicians, who, in addition to a lack of training, often lack practical experience in dealing with complex patients and challenging situations. At the same time, young physicians frequently form part of the care teams in family medicine practices. These factors can have various consequences, both for patient care and for the physicians themselves.

Furthermore, caring for complex patients can be emotionally exhausting, especially when dealing with incurable diseases or serious social issues. Family medicine physicians often have to make quick decisions without the opportunity to consult with colleagues. This is why they need to be highly clinically competent and confident in their own judgment, but also exposes them to significant stress (4).

Working in multidisciplinary teams that include nurses, social workers, psychologists, and other professionals can greatly enhance the care for complex patients and reduce the burden on individual health care providers. However, this approach requires clear communication, well-defined roles, and adequate organizational resources – the elements that are often lacking or insufficiently developed.

## System settings for managing complex patients in primary health care

Complex patients, with their specific profiles and multiple coexisting problems, are ideally suited to the competencies of family medicine physicians. It is precisely through the care of complex patients that the mission of family medicine is realized, as family medicine physicians respond to diverse patient needs and integrate various forms of care, from family and community support to the most advanced medical and social interventions. However, the fact that family medicine physicians possess the skills and a unique position to comprehensively address the needs of complex patients does not necessarily mean that they have the necessary conditions to provide such care. The key challenge lies in a mismatch between what family physicians can do for complex patients and what the health care system allows them to do in terms of adequate support and funding.

This leads to a paradox: the very patients who most need the care of family medicine physicians simultaneously pose the greatest burden for practices, which, without adequate resources, are left to handle these complex issues on their own (5). This situation threatens not only the quality of patient care but also the stability of the entire primary health care system, particularly by increasing the risk of physician burnout.

Therefore, caring for complex patients should be viewed as a priority not only for the sake of the patients and their families but also to ensure the sustainability of the primary health care system itself. The successful work of family medicine physicians and comprehensive community care cannot be achieved without systemic changes that include at least the adoption of new care models, allocation of adequate resources, improvements in medical education, and burnout prevention (6). Ultimately, caring for complex patients is not just a medical issue, it is also a matter of social justice and the sustainability of the health care system. By investing in support for family medicine physicians, we are laying the foundation for better care for all members of the community.
